# The Impact of Nutritional Components on Periodontal Health: A Literature Review

**DOI:** 10.3390/nu16223901

**Published:** 2024-11-15

**Authors:** Yarden Berg, Eran Gabay, Darko Božić, Jamil Awad Shibli, Ofir Ginesin, Thabet Asbi, Leila Takakura, Yaniv Mayer

**Affiliations:** 1 Department of Periodontology, School of Graduate Dentistry, Rambam Health Care Campus (RHCC), Haifa P.O. Box 9602, Israel; 2 Faculty of Medicine, Technion—Israel Institute of Technology, Haifa 3101101, Israel; 3 Department of Periodontology, School of Dental Medicine, HR-10000 Zagreb, Croatia; 4 Department of Periodontology, University Hospital Centre Zagreb, HR-10000 Zagreb, Croatia; 5 Department of Periodontology, Dental Research Division, Guarulhos University, Guarulhos 12245-000, SP, Brazil

**Keywords:** periodontal pathophysiology, nutritional modulation, micronutrient deficiency, anti-inflammatory nutrients, periodontal disease management

## Abstract

Periodontitis is a chronic inflammatory disease driven by the accumulation of bacterial plaque and the host’s immune response, leading to the destruction of periodontal tissues. Nutrition, particularly the intake of micronutrients with anti-inflammatory and antioxidant properties, plays a crucial role in maintaining periodontal health. This review explores the impact of various micronutrients—vitamins (A, B, C, D, E), minerals (calcium, iron, zinc, potassium, copper, manganese, selenium), and omega-3 fatty acids—on periodontal disease prevention and management. Deficiencies in these nutrients can exacerbate periodontal tissue damage by impairing immune responses, promoting oxidative stress, and reducing bone and tissue regeneration capabilities. While certain populations may be more vulnerable to these deficiencies, such as those following Western diets or living in low- and middle-income countries, even in developed nations, suboptimal nutrient intake is associated with worse periodontal outcomes. Although some studies suggest that supplementation of specific micronutrients may benefit periodontal therapy, the evidence remains inconclusive, necessitating further randomized clinical trials. This review underscores the importance of considering nutritional guidance in periodontal treatment protocols and highlights the need for tailored recommendations based on recent findings.

## 1. Introduction

Periodontitis is a chronic, inflammatory, and multifactorial disease caused by the buildup of bacterial plaque and characterized by the progressive destruction of the periodontal tissues. The main indicators of this condition include clinical attachment loss, radiographic bone loss, pocket depth, and gingival bleeding [1].

Periodontal tissue damage is primarily caused by the host’s improper response to bacterial plaque [2], which involves polymorphonuclear neutrophils (PMNs) attacking pathogens through intracellular signaling pathways and releasing free radicals. While free radicals are essential for neutralizing pathogens, an excess of them can be harmful to tissue cells, causing damage to DNA, lipids, and proteins [3].

While multiple factors influence oral microbiome balance and periodontal health, nutrition, especially consuming nutrients with anti-inflammatory and antioxidant properties, plays a significant role. A balanced diet can support the immune system, promote healing processes, reduce the risk of periodontal disease, and slow its progression. Studies have shown that populations that consume diets rich in antioxidants such as vitamins A, C, and E, fruits, vegetables, and omega-3 fatty acids demonstrate lower risks of developing periodontal disease [4]. In addition, sufficient vitamin D intake has been proven to improve periodontal health by reducing inflammation and promoting bone formation [5].

The Western diet, which includes high levels of pro-inflammatory components, processed foods, saturated fats, refined grains, sugar, fructose corn syrup, and alcohol, and low consumption of fruits and vegetables [6], can increase the risk of oxidative stress and contribute to a higher risk of periodontitis. Therefore, it is possible that monitoring the inflammatory component in nutrition may be a beneficial strategy for preventing and treating periodontal disease [7].

The Western diet may not provide all the necessary nutrients, but the problem is even more severe in low- and middle-income countries due to a lack of food sources rich in micronutrients such as grains, roots and tubers, legumes, nuts, dairy products, meat (beef, fish, poultry), eggs, fruits, and vegetables [8].

Nutritional components are divided into two categories:

1. Macronutrients: These are the main components that nourish and make up various tissues, serving as the primary source of calorie intake. They include carbohydrates, proteins, and fats [9].

2. Micronutrients: These do not significantly contribute to calorie intake but are important for maintaining health and essential functions, even when consumed in small quantities (µg/mg). This group includes vitamins (water-soluble or fat-soluble) and minerals necessary for various metabolic and physiological processes, inflammation regulation, immune system function, growth processes, maintenance of gum health, and prevention of periodontal diseases [10].

The impact of macronutrient deficiencies on periodontal health has been extensively studied. However, this review will specifically examine the potential impact of micronutrient deficiencies (excluding omega-3 fatty acids) on the development and progression of periodontal disease.

We conducted a comprehensive literature search across three major databases: PubMed, Scopus, and Web of Science. The search was designed to identify relevant studies published in peer-reviewed journals over the last 20 years, from January 2004 to December 2023. We focused on human studies to ensure clinical relevance and excluded animal research and studies without significant outcomes related to periodontal disease and nutrition.

The search terms were carefully selected to capture a broad range of research on the relationship between nutrition and periodontal health. We used the following keywords and phrases in various combinations:

- “Periodontal disease”;
- “Nutrition”;
- “Micronutrients”;
- “Vitamins” (including specific terms like “Vitamin A”, “Vitamin C”, “Vitamin D”, “Vitamin E”);
- “Minerals” (including terms like “Calcium,” “Zinc,” “Iron,” etc.);
- “Antioxidants”;
- “Omega-3 fatty acids”;
- “Dietary supplementation”;
- “Inflammation”;
- “Oxidative stress”.

Inclusion criteria comprised studies that explored the effects of specific nutrients on periodontal disease outcomes, discussed mechanisms underlying nutrient impact on periodontal health, or provided clinical insights into dietary modifications for periodontal patients. Exclusion criteria included animal studies, in vitro research, and articles without relevant clinical data.

This search strategy ensured a thorough and comprehensive review of the available literature, focusing on studies that offer clinical implications and mechanistic insights into the relationship between nutrition and periodontal disease. The recommended daily intake and main characteristics of each nutrient are presented in Table 1.

## 2. Omega-3 Fatty Acids

Omega-3 fatty acids, including eicosapentaenoic acid (EPA) and docosahexaenoic acid (DHA), are essential polyunsaturated fatty acids that must be obtained from food, such as fatty fish and plant-based sources. Omega-3 fatty acids are incorporated into the cell membranes [11], providing energy and serving as precursors for eicosanoids, which are signaling molecules with various roles in the cardiovascular, respiratory, immune, and endocrine systems [12].

Omega-3 supplements have been associated with potential anti-inflammatory effects and may contribute to the management of chronic inflammatory conditions [13], including periodontal health. Since EPA and DHA have anti-inflammatory properties, they may inhibit the activity of periodontal pathogens such as Porphyromonas gingivalis, Fusobacterium nucleatum, and Prevotella intermedia [11].

Research on the potential effects of omega-3 supplementation in periodontal treatment is extensive. Several studies [14,15,16,17] have evaluated the clinical impact of combining omega-3 supplements with non-surgical periodontal therapy, assessing the effects on periodontal probing depth (PD), clinical attachment loss (CAL), and inflammation indicators. The abovementioned studies [14,15,16,17] reported positive outcomes, including a meta-analysis that showed that in individuals with periodontitis, the use of omega-3 fatty acid dietary supplementation as an adjunct to non-surgical periodontal treatment can provide additional benefits in CAL gain and PPD reduction, compared with non-surgical periodontal treatment alone [14].

However, the evidence remains inconclusive, with some studies showing inconsistent results due to variations in omega-3 dosages, study durations, and outcomes across studies. Despite these limitations, the promising findings highlight the need for further randomized clinical trials with well-defined protocols to clarify the potential benefits of omega-3 in periodontal therapy [18]. It is important to note that while periodontal disease and tooth loss are linked to poor nutritional status, the evidence in the literature is characterized by high heterogeneity, resulting in varied confidence levels regarding these findings.

## 3. Vitamins

### 3.1. Vitamin A (Retinol)

Vitamin A is a group of fat-soluble vitamins, including retinol (found in animal products), beta-carotene (a form of vitamin A found in fruits and vegetables), and carotenoids. Retinol, the active form of vitamin A, is an antioxidant with anti-inflammatory properties. It helps inhibit inflammatory cytokines such as TNF-α, IL-6, and IL-12 [19]. Moreover, it has the ability to lead to dedifferentiation and pluripotency of cells [20], a critical feature for periodontal regeneration processes.

Vitamin A deficiency can be manifested in various ways, such as dry eyes (xerophthalmia), impaired corneal keratinization, night blindness, and, in severe cases, blindness. Oral side effects of vitamin A deficiency include dry mouth (xerostomia), increased risk of oral infections, and impaired tooth development in infants and children [21].

Several studies have shown that vitamin A deficiency is linked to an increased risk and severity of periodontal disease [22,23,24]. An exception is retinol, for which the association between its levels and periodontitis is not consistently supported by research. Some studies suggest that increased intake of beta-carotene (≥7.07 mg/day) is associated with a reduction in pocket depths greater than 3mm after scaling and root planning (SRP) [25]. Nevertheless, there is limited clinical evidence on the effects of vitamin A supplementation on periodontal healing and regeneration.

### 3.2. B Vitamins

Vitamin B is a complex of several water-soluble vitamins, including thiamine (B1), riboflavin (B2), niacin (B3), pantothenic acid (B5), pyridoxal (B6), biotin (B7), folic acid (B9), and cobalamin (B12). Each member of this family has a unique structure and function, contributing to various essential physiological processes such as nervous system development, DNA synthesis, red blood cell production, and amino acid metabolism [26].

Folic acid is an essential component in DNA repair processes and in preventing mutagenesis by promoting apoptosis (programmed cell death) of cells with DNA damage. Deficiency in folic acid has been linked to several diseases, including cardiovascular diseases, neural tube defects, and impaired wound healing [27]. In periodontal health, folic acid plays a critical role in the proliferation of epithelial cells. A deficiency in folic acid may increase the risk of periodontal disease progression by impairing cell turnover in the junctional epithelium (JE), which serves as a protective barrier. A deficiency of folic acid leads to the widening of intercellular spaces in this layer, negatively affecting its ability to act as a barrier and is a predisposing factor for infection of the oral mucosa, ulceration, gingivitis, periodontitis, and impaired wound healing [28].

Indeed, several studies have demonstrated more severe periodontal disease in patients with low serum levels of folic acid [29]. Another interesting finding is that serum folic acid concentrations among smoking patients with periodontal disease are significantly lower than those in non-smoking patients [30]. However, it has not yet been proven whether the administration of folic acid supplements helps prevent periodontal disease and improve treatment outcomes [29].

A recent study found an association between low serum B12 levels and increased clinical attachment loss (CAL) in patients with chronic periodontitis. Another study demonstrated that B12 levels were significantly lower in patients with chronic periodontitis than in healthy individuals [29].

### 3.3. Vitamin C (Ascorbic Acid)

Vitamin C is a water-soluble vitamin found in various fruits and vegetables. It is essential for collagen synthesis and immune function, and acts as an antioxidant to neutralize free radicals. In mammals, vitamin C serves as a cofactor for several enzymes involved in collagen formation, norepinephrine synthesis, and tyrosine metabolism. Its role as a reducing agent makes it essential for protecting cells from oxidative stress [31].

The interplay between vitamin C deficiency and periodontal pathology was described as early as the 18th century, when sailors suffered from scurvy, a deficiency of vitamin C associated with gum bleeding and tooth mobility caused by collagen structure changes and impaired connective tissue barrier formation [32]. Scurvy clinically manifests when blood levels of ascorbic acid fall below 11 µM, with standard ranges between 23 and 50 µM. While rare in developed countries, mild vitamin C deficiencies (serum levels below 24.8 µM/L) have been observed in approximately 10% of the general population [33] and about 30% of smokers [34].

Populations at higher risk for vitamin C deficiency include the elderly, infants exclusively fed on milk, alcoholics, and people who do not consume sufficient fruits and vegetables, with smokers being particularly vulnerable [35]. Patients with vitamin C deficiency demonstrate more significant clinical attachment loss (CAL) than those with normal levels [36], and there is a negative correlation between serum vitamin C levels and antibodies to periodontal pathogens like Aggregatibacter actinomycetemcomitans and *P. Gingivalis* [37].

Several studies have investigated the relationship between vitamin C levels and periodontal health [38], gaining a diverse understanding of the mechanisms that link vitamin C deficiency to periodontal disease:

1. A vitamin C deficiency can result in oxidative stress, which may subsequently cause tissue damage [39].
2. A vitamin C deficiency has been associated with gum bleeding regardless of oral hygiene levels [40].
3. A vitamin C supplementation and initial therapy have been shown to reduce gingival bleeding and inflammation in adult patients with gingivitis [41,42] and in diabetic patients [43].
4. However, it should be noted that in an interventional study, the addition of vitamin C alongside periodontal treatment did not reduce probing depths or clinical attachment gain for periodontitis [42].

### 3.4. Vitamin D [44]

Vitamin D is a fat-soluble vitamin primarily (90%) produced in the skin in response to sunlight. Small amounts, mostly D2 and D3 subtypes, are obtained from dietary sources such as fatty fish, egg yolks, and fortified dairy products. Once synthesized, vitamin D undergoes hydroxylation to form calcitriol, the active form of the vitamin. Calcitriol is critical in regulating calcium and phosphate metabolism, bone formation and remodeling, and immune responses [45].

A deficiency in vitamin D is defined as serum levels of its metabolite 25(OH)D3 falling below 10 nmol/L, with insufficiency being defined as levels between 21 and 29 nmol/L [46]. Vitamin D deficiency can have several consequences for oral health:

1. Periodontal Disease: Studies have shown that patients with periodontitis tend to have lower vitamin D levels compared to healthy individuals, regardless of smoking status. This association has been significant particularly among women with moderate periodontitis, but it exists at all stages of periodontal disease (from gingivitis to chronic periodontitis) [47].
2. Bone Loss: Vitamin D deficiency contributes to the progression of alveolar bone resorption, which can lead to tooth loss [47].
3. Impaired Wound Healing: Low vitamin D levels can impair the healing process following periodontal surgery [47,48].
4. Reduced Antibacterial Defense: Vitamin D enhances the antibacterial defense of gingival epithelial cells. A vitamin D deficiency may impair this protective mechanism, thus increasing susceptibility to oral infections [47].
5. Inflammation: Vitamin D deficiency has been associated with increased levels of gingivitis, which may contribute to the further progression of periodontal diseases [47].

Vitamin D also exerts immunomodulatory effects by regulating the production of cytokines and chemokines, as well as the activity of immune cells like T and B lymphocytes [49]. This helps balance the immune response and prevent excessive inflammation in the gingival tissues. In addition, vitamin D has anti-inflammatory effects by reducing the production of pro-inflammatory cytokines such as TNF-α and IL-6 [47].

### 3.5. Vitamin E

Vitamin E is a group of eight fat-soluble compounds, with alpha-tocopherol being the most biologically active form. It acts as an antioxidant that protects cell membranes from oxidative damage [30].

A systematic review of studies investigating the impact of alpha-tocopherol on periodontal health [50] found that higher serum levels of this form of vitamin E were inversely correlated with average probing pocket depth (PPD) in patients with periodontitis, while gamma-tocopherol showed a moderate association among patients who demonstrated a lower average PPD compared to their counterparts with a higher average.

Furthermore, a similar study that used data from over 15,000 patients who participated in the NHANES cohort studies showed that the relationship between vitamin levels and periodontal health exists across all severity levels of periodontitis (even though statistical significance was only achieved for moderate periodontitis) and that the relationship is not dependent on ethnic origin, although individuals with darker skin consistently exhibited lower levels of vitamin E [30].

In contrast to these articles, it should be noted that no relationship was found between the two forms of vitamin E and periodontitis among patients in the PRIME cross-sectional study [51].

Moreover, excessive intake of certain micronutrients can be detrimental to periodontal health. For example, excess vitamin D may cause hypercalcemia, which negatively impacts bone metabolism. Thus, it is essential to monitor the intake of micronutrient supplements carefully.

## 4. Minerals

### 4.1. Calcium

Calcium is vital in maintaining oral health, particularly in bone and tooth formation and maintenance. About 99% of the body’s calcium is stored in bones and teeth (primarily in its form as hydroxyapatite), while the remaining 1% is found in soft tissues and body fluids.

Calcium is crucial in extracellular and intracellular environments, influencing various cellular processes and signaling pathways. Its extracellular level is constant (2.2–2.6 mmol/L) and is maintained by three hormones (parathyroid hormone, calcitonin, and 1,25-dihydroxycholecalciferol), which aim to preserve homeostasis and support vital functions such as bone mineralization and muscle contraction. The intracellular concentration of calcium is also tightly regulated and plays a central role in regulating cell membrane permeability, hormone and neurotransmitter release for signaling and intercellular communication, nucleotide formation and breakdown, and many other processes that assist in regulating events such as proliferation, differentiation, and apoptosis [52].

In the context of inflammation, intracellular signaling processes that utilize calcium activate immune cells and produce inflammatory mediators in response to microbial challenges in periodontal tissues. Inflammatory stimulation can increase calcium levels in immune system cells, thereby activating signaling pathways that promote the production of pro-inflammatory cytokines, chemokines, and oxidants. These inflammatory mediators contribute to the recruitment of immune cells to locate the infection or injury and enhance the inflammatory response.

In the context of periodontal disease, a lack of regulation in calcium homeostasis may damage the periodontal tissues due to excessive inflammatory activity in response to periodontal pathogens. Studies have shown that adequate calcium intake is associated with a nearly 20% lower incidence of periodontitis compared to individuals with low calcium intake, as well as a reduced risk of progression of existing disease and loss of attachment (regardless of other significant risk factors such as smoking, blood sugar levels, and BoP) [53]. Furthermore, serum calcium levels have been inversely associated with the progression of periodontal disease in older populations. Lastly, low calcium intake has been shown to increase the risk of tooth loss in both men and women, highlighting the importance of maintaining adequate calcium levels for gum health [54].

Regarding the impact on periodontal treatment, calcium supplements have demonstrated promising results in improving treatment outcomes: calcium intake, whether through dietary sources or supplements, has been linked to a lower risk of tooth loss. Studies have indicated that taking calcium supplements during specific periods and adequate calcium intake may positively affect long-term tooth preservation [54].

Moreover, when examining the effect of vitamin D and calcium supplementation in patients undergoing periodontal maintenance therapy, those who took a vitamin D supplement (≥400 IU) and calcium (≥1000 mg) for an average of 10 years showed improved clinical periodontal parameters compared to their counterparts who did not take dietary supplements (even at a borderline statistical significance). Among these parameters were pocket depth, number of sites with bleeding, gingival index, probing depth, loss of attachment, and loss of radiographic bone support [29].

### 4.2. Iron

Iron is a critical component of hemoglobin and various enzymes involved in metabolic processes. Iron deficiency can lead to anemia, which may contribute to oxidative stress and an imbalance between reactive oxygen species and antioxidants in the body, both of which can negatively affect periodontal health [55].

Beyond its systemic benefits, iron is also important for maintaining gum health, and a deficiency can impair the body’s ability to fight periodontal pathogens. For example, patients with anemia caused by iron deficiency who also suffer from chronic periodontitis have shown a higher frequency of bleeding upon probing, a greater percentage of sites with clinical attachment loss of 6 mm or greater, and deeper pockets compared to those with chronic periodontitis alone [56].

Additionally, in a case–control study involving periodontal patients with and without iron deficiency anemia, researchers found that following non-surgical periodontal treatment, serum levels of iron, ferritin, and hemoglobin increased in the anemia group [57]. While the systemic effects of iron are well known, further research is needed to determine the specific benefits of iron supplements as an adjunct to periodontal treatment and their potential impact on the progression of periodontal diseases.

### 4.3. Zinc

Zinc is an essential mineral for the proper functioning of the human body. It is a crucial component of many enzymes involved in various biological processes. Zinc aids in the body’s growth and development, plays a vital role in wound healing, and is necessary for cell membrane stability. It acts as a cofactor for over 300 enzymes, helping to regulate the synthesis and degradation of essential biomolecules such as fats, proteins, and nucleic acids, contributes to immune function, possesses antioxidant properties, and supports various metabolic processes [58].

Zinc is also essential for maintaining gum health: it plays a significant role in supporting enzymatic activities, facilitates proper protein folding, and helps maintain the structural integrity of periodontal tissues. Zinc deficiency can contribute to the development of gingival inflammation through various mechanisms [59]:

1. Immunological Response: Zinc deficiency can impair immune system function, reducing its ability to combat infections, including those in the oral cavity.
2. Oxidative Stress: A lack of zinc can increase oxidative stress in the body, which is known to play a role in the development and progression of gum diseases.
3. Antioxidant Properties: Zinc has antioxidant properties that help protect cells from damage caused by oxidizing agents. Consequently, a decrease in zinc levels may lead to reduced protective effects.
4. Enzymatic Regulation: Zinc is essential for the activity of many enzymes in the body, and zinc deficiency can disrupt enzymatic regulation processes crucial for maintaining oral health.
5. Bone Regeneration: Zinc is vital for bone regeneration processes. A deficiency can hinder the body’s ability to repair and maintain bone tissue in the oral cavity, which is essential for oral health. Low zinc levels have been associated with increased alveolar bone resorption in patients with periodontitis, through a mechanism involving elevated levels of enzymes like phosphatase, which are related to destructive bone processes occurring during advanced stages of periodontitis [60].
6. Periodontal Treatment Effects: Studies have shown that non-surgical periodontal treatment positively impacts serum zinc concentrations: three months after treatment in patients with chronic periodontitis, both with and without diabetes, serum zinc levels increased significantly compared to baseline values across all study groups [61].

### 4.4. Potassium

Potassium is the primary intracellular cation in the body and plays a crucial role in various cellular functions such as metabolism, growth, enzyme activity, and DNA synthesis. The distribution of potassium between the intracellular and extracellular compartments is influenced by various factors such as insulin, basic pH (alkalosis), and catecholamines, which enhance the entry of potassium into cells. About 90% of dietary potassium is absorbed in the digestive system and is eventually excreted in urine, thus maintaining potassium balance in the body. Changes in potassium concentration can negatively affect health, with low potassium levels in the blood (hypokalemia) causing symptoms such as muscle weakness, slowed intestinal activity, and cardiac rhythm disturbances [24].

A study by Yamori et al. found a significant relationship between potassium intake and the severity of gum inflammation among middle-aged Tanzanian women. The study demonstrated that low potassium intake, often accompanied by a deficiency in dietary fiber, may not only exacerbate gum inflammation but also elevate blood pressure [62].

### 4.5. Copper

One of copper’s main characteristics in biological systems is its ability to exist in both reduced (Cu^+^) and oxidized (Cu^2+^) states, allowing it to participate in a wide range of biochemical reactions. Copper-containing proteins play a crucial role in maintaining cellular redox balance and are, therefore, important for immune system function [55].

Studies in southwestern India have shown elevated copper levels in subjects with periodontitis, particularly those with diabetes, compared to individuals without gum inflammation [63,64]. Furthermore, a recent study indicated that copper levels in patients with chronic gingivitis, both with and without diabetes, significantly improved after non-surgical periodontal treatment [61].

### 4.6. Manganese

Manganese is involved in metabolic processes, bone formation, the reproductive system, and the immune system. Additionally, manganese works together with vitamin K for blood clotting. Accordingly, manganese deficiency reduces bone density and bone formation.

A study by Kim HS et al., 2014, showed an inverse relationship between plasma manganese levels and the periodontal status of the participants, even after adjusting for socio-demographic variables, oral hygiene habits, oral health status, and systemic background conditions [65,66].

### 4.7. Selenium

Selenium is primarily consumed through plant sources. The bioavailability of selenium from these food sources is influenced by its chemical form: selenomethionine, a form of selenium amino acids, is a major source of selenium metabolized by humans. This form, along with inorganic selenium salts, can be converted through metabolism into selenoproteins, essential for various enzymatic activities in the body.

Since the selenium level in vegetation depends on the concentration in the surrounding soil, selenium consumption levels vary across different geographical areas. The human body absorbs the mineral most effectively in the presence of fat-soluble vitamins such as A, D, and E. Selenium has several vital roles, including antioxidant, anti-inflammatory, and antiviral properties. Additionally, studies have shown that selenium plays a crucial role in cellular maintenance, protein folding, and the body’s response to oxidative stress. Changes in selenium levels or selenoprotein activity have also been linked to the development of chronic diseases, including cancer [67].

In a study by Thomas et al., 2013, researchers found that plasma selenium concentrations were lower in middle-aged subjects with type 2 diabetes and periodontitis compared to their healthy counterparts [64].

Here is a table summarizing the various micronutrients discussed, including the recommended daily intake, dietary sources, and their importance for overall and periodontal health (Table 1):

**Table 1:** Summary of micronutrients and their influence on periodontal health.

Micronutrient	Dietary Source	Importance for General Health	Importance for Periodontal Health	Recommended Daily Intake (for Adults, According to NHS)	References
Vitamin A	Cheese, milk, yogurt, eggs, fish oil, liver. β-carotene: yellow, red, and green vegetables (spinach, carrots, sweet potatoes). Yellow fruits (mango, papaya, apricot).	Essential for immune system regulation, vision, and cell division, and acts as an antioxidant. Its role is unclear; further research is needed.	Several studies have shown that deficiency is linked to an increased risk and severity of periodontal disease. Increased β-carotene linked to improvement after SRP following CAL.	Men—700 μg/day Women—600 μg/day	[22,23,24,25]
Vitamin B9 (Folic Acid) + Vitamin B12	B9—broccoli, Brussels sprouts, leafy vegetables (cabbage, kale, spinach). Peas, chickpeas, beans, liver, cereals. B12—cereals, meat, fish, dairy, cheese, eggs.	Development of the nervous system (reduces risk of defects like spina bifida), DNA synthesis, red blood cell formation, amino acid metabolism.	Folic acid insufficiency has previously been associated with chronic periodontitis in smokers; Previous studies have shown that smokers with periodontal disease tend to have lower folic acid levels	B9—200 μg/day B12—1.5 μg/day	[26,29,30,68]
Vitamin C	Citrus fruits, peppers, strawberries, broccoli, potatoes, Brussels sprouts.	Maintains skin, blood vessels, bones, and cartilage health. Crucial for wound healing.	Bleeding and inflammation of gums are symptoms of scurvy. Supplementation may improve periodontal treatment outcomes.	40 mg/day	[31,32,33,34,35,36,37,39,40,41,42,43]
Vitamin D	Primarily from sunlight. Small amounts in fish (salmon, sardines, mackerel), red meat, liver, and egg yolk.	Sufficient sunlight exposure can provide the necessary dose; the recommended supplement is about 10 µg/day.	Helps regulate calcium and phosphate levels. Deficiency may delay healing or osseointegration after surgical procedures.	While the required dosage can be obtained from sufficient exposure to sunlight, it is recommended to take a supplement of about 10 µg/day.	[45,46,47,69]
Vitamin E	Vegetable oils (soybean, olive, sunflower, corn). Nuts, wheat germ.		Deficiency may lead to gum bleeding. The effect of consumption on periodontal treatment outcomes is unknown.	Men—4 mg/day Women—3 mg/day	[30,50,51]
Calcium	Dairy products (milk, cheese, yogurt), leafy green vegetables (kale, broccoli), almonds, tofu, fortified foods (orange juice, cereals).	Essential for bone health, muscle function, nerve signaling, and blood clotting.	Plays a crucial role in maintaining the integrity of the periodontal tissue and supports bone health in the jaw.	700 mg/day	[53,54,70]
Iron	Red meat, poultry, fish, lentils, beans, fortified cereals, spinach.	Vital for producing hemoglobin, which carries oxygen in the blood and supports immune function.	Adequate iron levels are essential for reducing inflammation and promoting healing in periodontal tissues.	Men—8.7 mg/day Women under the age of 50—14.8 mg/day Women who are 50 years old or older—8.7 mg/day	[55,56,57]
Zinc	Meat, shellfish, legumes, seeds, nuts, dairy, whole grains.	Supports immune function, wound healing, and protein synthesis; essential for DNA synthesis and cell division.	Important for tissue repair and regeneration; deficiency may lead to increased susceptibility to periodontal disease.	Men—9.5 mg/day Women—7 mg/day	[58,59,60,61]
Potassium	Leafy green vegetables, oranges and grapefruits, potatoes, and bananas.	Essential for metabolic processes, growth, enzyme activity, and DNA synthesis.	Low intake may worsen gum inflammation.	Men—3400 mg/day Women—2600 mg/day	[24,62]
Copper	Liver, shellfish, nuts, mushrooms, whole grains, and dried legumes.	Maintains cellular redox balance.	High levels were found in diabetic patients with periodontitis when compared to healthy individuals with periodontitis.	1.2 mg/day	[24,63]
Manganese	Whole grains, legumes, leafy greens, avocados, nuts.	Metabolic processes, bone formation, immune system function.	An inverse relationship was found between plasma manganese levels and periodontal status.	Men—2.3 mg/day Women—1.8 mg/day	[65,66]
Selenium	Grains, seafood, meat, eggs, dairy products, yeast, and nuts.	Cellular maintenance, protein folding, and the body’s response to oxidative stress.	Low levels found in subjects with periodontitis compared to healthy subjects.	55 mcg/day	[66,67]

## 5. Discussion

Our review has highlighted significant heterogeneity among studies assessing the impact of nutrition on periodontal health. This variability stems from differences in study design, population characteristics, outcome measures, and assessment methods. Consequently, the quality of evidence remains low, and standardized research protocols are urgently needed to draw more reliable conclusions.

The relationship between nutrition and periodontal health is increasingly recognized as a significant factor influencing the development, progression, and prevention of periodontitis [4]. This review has synthesized current findings on various micronutrients, including vitamins A, C, D, and E, and essential minerals, elucidating their roles in periodontal disease. The evidence presented highlights the multifaceted nature of periodontal health, demonstrating how deficiencies in these nutrients can exacerbate inflammatory responses, impair immune function, and contribute to tissue degradation [4,5,22].

One of the most compelling findings from this review is the significant association between vitamin C deficiency and periodontal disease severity. Vitamin C is crucial for collagen synthesis and vital for maintaining periodontal tissue’s structural integrity [38]. A systematic review indicated that individuals with lower plasma concentrations of vitamin C exhibited increased probing depths and clinical attachment loss, suggesting that inadequate vitamin C levels may lead to compromised periodontal tissue health [36,37,38,45,46], reinforcing the need for adequate dietary intake of this essential nutrient.

Similarly, vitamin D has emerged as a key player in modulating inflammatory responses and promoting bone health. While some studies have linked vitamin D deficiency to the progression of alveolar bone resorption [47], others highlighted the observed increase in gingivitis levels as it enhances the antibacterial defense of gingival epithelial cells [71]. Understanding the data is complicated due to the heterogeneity of studies, which have used different case definitions for diagnosing periodontitis and various thresholds for vitamin D deficiency and sufficiency [44].

This review also highlights vitamin E’s role in periodontal health, particularly alpha-tocopherol. A systematic review found that higher serum levels of vitamin E were inversely correlated with probing pocket depth in patients with periodontitis, suggesting that this antioxidant may play a protective role against periodontal tissue destruction [50]. The antioxidant properties of vitamin E help mitigate oxidative stress, which is known to contribute to periodontal inflammation and tissue damage. However, it is noteworthy that some studies, such as the PRIME cross-sectional study, did not find a significant relationship between vitamin E levels and periodontitis [51]. This indicates the need for further investigation to clarify these discrepancies and fully understand vitamin E’s role in periodontal health.

In addition to vitamins, this review emphasizes the importance of essential minerals such as calcium, zinc, and iron in periodontal health. Calcium is vital for maintaining bone density and structure [52], and its deficiency can lead to increased bone resorption, exacerbating periodontal disease [53]. Zinc, known for its role in immune function and wound healing, has been shown to have a protective effect against periodontal disease progression. Studies indicate that zinc deficiency may impair the host’s immune response to periodontal pathogens, thereby increasing susceptibility to infection [59]. Iron, while essential for various physiological functions, has also been implicated in periodontal disease, with deficiencies leading to compromised immune responses and increased inflammation [55].

This review further underscores the potential benefits of omega-3 fatty acids in periodontal health. Omega-3 fatty acids, particularly eicosapentaenoic acid (EPA) and docosahexaenoic acid (DHA), possess anti-inflammatory properties that may inhibit the activity of periodontal pathogens such as Porphyromonas gingivalis [11]. Research has shown that supplementation with omega-3 fatty acids can lead to significant improvements in clinical parameters, including probing depth and clinical attachment loss, when used as an adjunct to non-surgical periodontal therapy [14]. These findings suggest that dietary interventions incorporating omega-3 fatty acids could serve as a valuable strategy in managing periodontal disease. Despite the evidence being inconclusive, some studies have shown inconsistent results owing to variations in omega-3 dosages, study lengths, and outcomes [18].

The implications of these findings extend beyond individual nutrient deficiencies. The review highlights the detrimental effects of the Western diet, characterized by high levels of processed foods and low nutrient density, on periodontal health [6]. This dietary pattern not only contributes to micronutrient deficiencies but also promotes systemic inflammation, thereby increasing the risk of periodontitis [7]. In contrast, populations adhering to diets rich in antioxidants and anti-inflammatory components, such as fruits, vegetables, and omega-3 sources, exhibit lower incidences of periodontal disease [4], while a whole-food plant-based diet has been shown to have a trend towards stabilization of inflammatory parameters (BOP, PISA [periodontal inflamed surface area]) in patients with increased cardiometabolic risk [72]. These correlations emphasize the need for public health initiatives aimed at improving dietary habits as a preventive strategy against periodontal disease.

Periodontal disease is complex and influenced by numerous factors beyond inadequate nutrition. These include genetic predisposition, socio-economic status, lifestyle behaviors (such as smoking and alcohol consumption), and underlying systemic conditions. Adequate oral care remains essential; however, these additional factors may significantly modify disease risk and progression.

Moreover, this review calls attention to the necessity of integrating nutritional assessments and interventions into periodontal treatment protocols. Current guidelines often overlook the role of nutrition, despite the growing body of evidence supporting its significance. By incorporating nutritional evaluations into clinical practice, healthcare providers can identify individuals at risk for deficiencies and tailor dietary recommendations accordingly. This approach not only addresses the underlying nutritional deficiencies that may hinder healing but also empowers patients to take an active role in their periodontal health.

Despite promising findings for some micronutrient interventions, the evidence is far from conclusive. Studies vary significantly in dosages, supplementation duration, and clinical outcomes, and in some cases, no consistent improvement in periodontal parameters is observed. This inconsistency underscores the need for more robust, randomized clinical trials to better determine the efficacy of specific nutrient supplements.

Our review contributes to the existing literature by providing a comprehensive synthesis of the current evidence on micronutrient impacts on periodontal health. The strength of this review lies in its extensive scope, covering multiple nutrients and their interactions with periodontal disease mechanisms. However, limitations include reliance on observational studies with inherent biases and inconsistencies, and the overall heterogeneity of methodologies that challenge the establishment of definitive conclusions.

Drawing from the insights of this review, we recommend specific dietary guidelines for managing periodontal disease. Patients are advised to consume a diet rich in anti-inflammatory and antioxidant nutrients, such as vitamins A, C, D, and E, alongside omega-3 fatty acids. Prioritizing foods like leafy green vegetables, fatty fish (e.g., salmon), nuts, and seeds, while reducing the intake of processed and sugary items, can promote periodontal health by modulating immune functions and mitigating oxidative stress. When serum levels of essential micronutrients fall below critical thresholds—for example, vitamin D under 30 ng/mL or vitamin C below 0.2 mg/dL—supplementation may become necessary. These values are of particular clinical relevance for individuals at heightened risk of periodontal disease.

## 6. Conclusions

In conclusion, the findings of this review contribute to a deeper understanding of the role of micronutrients in periodontal health. They underscore the necessity for further research to elucidate the specific mechanisms through which these nutrients influence periodontal disease processes. Future studies should aim to establish clear guidelines for dietary recommendations and supplementation strategies tailored to individuals at risk for or suffering from periodontitis. By fostering a comprehensive approach that includes nutritional considerations, we can enhance the prevention and management of periodontal disease, ultimately improving patient outcomes and quality of life. Given the multifactorial nature of periodontal disease, a multidisciplinary treatment approach is essential. Integrating a nutritionist into the healthcare team can provide comprehensive dietary guidance, address nutrient deficiencies, and potentially improve patient outcomes. Future research should focus on well-structured, high-quality studies to establish clearer dietary recommendations and investigate synergistic effects of nutrition and other therapies.

## Data Availability

No new data were created or analyzed in this study.

## References

[1] Papapanou P.N., Sanz M., Buduneli N., Dietrich T., Feres M., Fine D.H., Flemmig T.F., Garcia R., Giannobile W.V., Graziani F. (2018). Periodontitis: Consensus report of workgroup 2 of the 2017 World Workshop on the Classification of Periodontal and Peri-Implant Diseases and Conditions. J. Periodontol..

[2] Bartold P.M., Van Dyke T.E. (2013). Periodontitis: A host-mediated disruption of microbial homeostasis. Unlearning learned concepts. Periodontology 2000.

[3] Kanzaki H., Wada S., Narimiya T., Yamaguchi Y., Katsumata Y., Itohiya K., Fukaya S., Miyamoto Y., Nakamura Y. (2017). Pathways that Regulate ROS Scavenging Enzymes, and Their Role in Defense Against Tissue Destruction in Periodontitis. Front. Physiol..

[4] Santonocito S., Polizzi A., Palazzo G., Indelicato F., Isola G. (2021). Dietary Factors Affecting the Prevalence and Impact of Periodontal Disease. Clin. Cosmet. Investig. Dent..

[5] Varela-López A., Navarro-Hortal M.D., Giampieri F., Bullón P., Battino M., Quiles J.L. (2018). Nutraceuticals in Periodontal Health: A Systematic Review on the Role of Vitamins in Periodontal Health Maintenance. Molecules.

[6] Uranga J.A., López-Miranda V., Lombó F., Abalo R. (2016). Food, nutrients and nutraceuticals affecting the course of inflammatory bowel disease. Pharmacol. Rep..

[7] Sczepanik F.S.C., Grossi M.L., Casati M., Goldberg M., Glogauer M., Fine N., Tenenbaum H.C. (2020). Periodontitis is an inflammatory disease of oxidative stress: We should treat it that way. Periodontology 2000.

[8] Engidaw M.T., Gebremariam A.D., Tiruneh S.A., Tesfa D., Fentaw Y., Kefale B., Tiruneh M., Wubie A.T. (2023). Micronutrient intake status and associated factors in children aged 6-23 months in sub-Saharan Africa. Sci. Rep..

[9] Carreiro A.L., Dhillon J., Gordon S., Higgins K.A., Jacobs A.G., McArthur B.M., Redan B.W., Rivera R.L., Schmidt L.R., Mattes R.D. (2016). The Macronutrients, Appetite, and Energy Intake. Annu. Rev. Nutr..

[10] Najeeb S., Zafar M.S., Khurshid Z., Zohaib S., Almas K. (2016). The Role of Nutrition in Periodontal Health: An Update. Nutrients.

[11] Chee B., Park B., Fitzsimmons T., Coates A.M., Bartold P.M. (2016). Omega-3 fatty acids as an adjunct for periodontal therapy—A review. Clin. Oral Investig..

[12] Jones P.J.H., Papamandjaris A.A., Erdman J.W., Macdonald I.A., Zeisel S.H. (2012). Lipids: Cellular Metabolism. Present Knowledge in Nutrition.

[13] Chatterjee D., Chatterjee A., Kalra D., Kapoor A., Vijay S., Jain S. (2021). Role of adjunct use of omega 3 fatty acids in periodontal therapy of periodontitis. A systematic review and meta-analysis. J. Oral Biol. Craniofacial Res..

[14] Castro Dos Santos N.C., Furukawa M.V., Oliveira-Cardoso I., Cortelli J.R., Feres M., Van Dyke T., Rovai E.S. (2022). Does the use of omega-3 fatty acids as an adjunct to non-surgical periodontal therapy provide additional benefits in the treatment of periodontitis? A systematic review and meta-analysis. J. Periodontal Res..

[15] Castro Dos Santos N.C., Andere N.M.R.B., Araujo C.F., de Marco A.C., Kantarci A., Van Dyke T.E., Santamaria M.P. (2020). Omega-3 PUFA and aspirin as adjuncts to periodontal debridement in patients with periodontitis and type 2 diabetes mellitus: Randomized clinical trial. J. Periodontol..

[16] Miller L.M., Piccinin F.B., van der Velden U., Gomes S.C. (2022). The Impact of Omega-3 Supplements on Non-Surgical Periodontal Therapy: A Systematic Review. Nutrients.

[17] Maybodi F.R., Fakhari M., Tavakoli F. (2022). Effects of omega-3 supplementation as an adjunct to non-surgical periodontal therapy on periodontal parameters in periodontitis patients: A randomized clinical trial. BMC Oral Health.

[18] Kruse A.B., Kowalski C.D., Leuthold S., Vach K., Ratka-Krüger P., Woelber J.P. (2020). What is the impact of the adjunctive use of omega-3 fatty acids in the treatment of periodontitis? A systematic review and meta-analysis. Lipids Health Dis..

[19] Kim S.Y., Koo J.E., Song M.R., Lee J.Y. (2013). Retinol suppresses the activation of Toll-like receptors in MyD88- and STAT1-independent manners. Inflammation.

[20] Hore T.A. (2017). Modulating epigenetic memory through vitamins and TET: Implications for regenerative medicine and cancer treatment. Epigenomics.

[21] Schlosser B.J., Pirigyi M., Mirowski G.W. (2011). Oral manifestations of hematologic and nutritional diseases. Otolaryngol. Clin. N. Am..

[22] Luo P.P., Xu H.S., Chen Y.W., Wu S.P. (2018). Periodontal disease severity is associated with micronutrient intake. Aust. Dent. J..

[23] Mi N., Zhang M., Ying Z., Lin X., Jin Y. (2024). Vitamin intake and periodontal disease: A meta-analysis of observational studies. BMC Oral Health.

[24] Dommisch H., Kuzmanova D., Jönsson D., Grant M., Chapple I. (2018). Effect of micronutrient malnutrition on periodontal disease and periodontal therapy. Periodontol. 2000.

[25] Dodington D.W., Fritz P.C., Sullivan P.J., Ward W.E. (2015). Higher Intakes of Fruits and Vegetables, β-Carotene, Vitamin C, α-Tocopherol, EPA, and DHA Are Positively Associated with Periodontal Healing after Nonsurgical Periodontal Therapy in Nonsmokers but Not in Smokers. J. Nutr..

[26] Allen L.H. (2012). Vitamin B-12. Adv. Nutr..

[27] Keceli H.G., Ercan N., Karsiyaka Hendek M., Kisa U., Mesut B., Olgun E. (2020). The effect of the systemic folic acid intake as an adjunct to scaling and root planing on clinical parameters and homocysteine and C-reactive protein levels in gingival crevicular fluid of periodontitis patients: A randomized placebo-controlled clinical trial. J. Clin. Periodontol..

[28] Vogel R.I., Fink R.A., Frank O., Baker H. (1978). The effect of topical application of folic acid on gingival health. J. Oral Med..

[29] Hans M., Malik P.K., Hans V.M., Chug A., Kumar M. (2023). Serum levels of various vitamins in periodontal health and disease—A cross sectional study. J. Oral Biol. Craniofacial Res..

[30] Ebersole J.L., Lambert J., Bush H., Huja P.E., Basu A. (2018). Serum Nutrient Levels and Aging Effects on Periodontitis. Nutrients.

[31] Frei B., Birlouez-Aragon I., Lykkesfeldt J. (2012). Authors’ perspective: What is the optimum intake of vitamin C in humans?. Crit. Rev. Food Sci. Nutr..

[32] Glickman I. (1948). Acute vitamin C deficiency and the periodontal tissues; the effect of acute vitamin C deficiency upon the response of the periodontal tissues of the guinea pig to artificially induced inflammation. J. Dent. Res..

[33] Lindblad M., Tveden-Nyborg P., Lykkesfeldt J. (2013). Regulation of vitamin C homeostasis during deficiency. Nutrients.

[34] Schectman G., Byrd J.C., Hoffmann R. (1991). Ascorbic acid requirements for smokers: Analysis of a population survey. Am. J. Clin. Nutr..

[35] Nishida M., Grossi S.G., Dunford R.G., Ho A.W., Trevisan M., Genco R.J. (2000). Dietary vitamin C and the risk for periodontal disease. J. Periodontol..

[36] Amaliya A., Laine M.L., Delanghe J.R., Loos B.G., Van Wijk A.J., Van der Velden U. (2015). Java project on periodontal diseases: Periodontal bone loss in relation to environmental and systemic conditions. J. Clin. Periodontol..

[37] Pussinen P.J., Laatikainen T., Alfthan G., Asikainen S., Jousilahti P. (2003). Periodontitis is associated with a low concentration of vitamin C in plasma. Clin. Vaccine Immunol..

[38] Van der Velden U., Kuzmanova D., Chapple I.L. (2011). Micronutritional approaches to periodontal therapy. J. Clin. Periodontol..

[39] Chapple I.L., Matthews J.B. (2007). The role of reactive oxygen and antioxidant species in periodontal tissue destruction. Periodontology 2000.

[40] Hujoel P.P., Lingström P. (2017). Nutrition, dental caries and periodontal disease: A narrative review. J. Clin. Periodontol..

[41] Shimabukuro Y., Nakayama Y., Ogata Y., Tamazawa K., Shimauchi H., Nishida T., Ito K., Chikazawa T., Kataoka S., Murakami S. (2015). Effects of an ascorbic acid-derivative dentifrice in patients with gingivitis: A double-masked, randomized, controlled clinical trial. J. Periodontol..

[42] Fageeh H.N., Fageeh H.I., Prabhu A., Bhandi S., Khan S., Patil S. (2021). Efficacy of vitamin C supplementation as an adjunct in the non-surgical management of periodontitis: A systematic review. Syst. Rev..

[43] Gokhale N.H., Acharya A.B., Patil V.S., Trivedi D.J., Thakur S.L. (2013). A short-term evaluation of the relationship between plasma ascorbic acid levels and periodontal disease in systemically healthy and type 2 diabetes mellitus subjects. J. Diet. Suppl..

[44] Lu E.M. (2023). The role of vitamin D in periodontal health and disease. J. Periodontal Res..

[45] Colotta F., Jansson B., Bonelli F. (2017). Modulation of inflammatory and immune responses by vitamin D. J. Autoimmun..

[46] Makke A. (2022). Vitamin D Supplementation for Prevention of Dental Implant Failure: A Systematic Review. Int. J. Dent..

[47] Amano Y., Komiyama K., Makishima M. (2009). Vitamin D and periodontal disease. J. Oral Sci..

[48] Bashutski J.D., Eber R.M., Kinney J.S., Benavides E., Maitra S., Braun T.M., Giannobile W.V., McCauley L.K. (2011). The impact of vitamin D status on periodontal surgery outcomes. J. Dent. Res..

[49] Jagelavičienė E., Vaitkevičienė I., Šilingaitė D., Šinkūnaitė E., Daugėlaitė G. (2018). The Relationship between Vitamin D and Periodontal Pathology. Medicina.

[50] Barnawi B.M., Alrashidi N.S., Albalawi A.M., Alakeel N.S., Hamed J.T., Barashid A.A., Alduraibi M.S., Alhussain G.S., Alghadeer J.Y., Alarifi N.A. (2023). Nutritional Modulation of Periodontal Diseases: A Narrative Review of Recent Evidence. Cureus.

[51] Linden G.J., McClean K.M., Woodside J.V., Patterson C.C., Evans A., Young I.S., Kee F. (2009). Antioxidants and periodontitis in 60-70-year-old men. J. Clin. Periodontol..

[52] Sukumaran P., Nascimento Da Conceicao V., Sun Y., Ahamad N., Saraiva L.R., Selvaraj S., Singh B.B. (2021). Calcium Signaling Regulates Autophagy and Apoptosis. Cells.

[53] Al-Zahrani M.S. (2006). Increased intake of dairy products is related to lower periodontitis prevalence. J. Periodontol..

[54] Krall E.A., Wehler C., Garcia R.I., Harris S.S., Dawson-Hughes B. (2001). Calcium and vitamin D supplements reduce tooth loss in the elderly. Am. J. Med..

[55] Li Y., Du Y., Zhou Y., Chen Q., Luo Z., Ren Y., Chen X., Chen G. (2023). Iron and copper: Critical executioners of ferroptosis, cuproptosis and other forms of cell death. Cell Commun. Signal..

[56] Chakraborty S., Tewari S., Sharma R.K., Narula S.C., Ghalaut P.S., Ghalaut V. (2014). Impact of iron deficiency anemia on chronic periodontitis and superoxide dismutase activity: A cross-sectional study. J. Periodontal Implant. Sci..

[57] Enhos S., Duran I., Erdem S., Buyukbas S. (2009). Relationship between iron-deficiency anemia and periodontal status in female patients. J. Periodontol..

[58] Stiles L.I., Ferrao K., Mehta K.J. (2024). Role of zinc in health and disease. Clin Exp Med..

[59] Zhou F., Yao S., Shan F., Zhou Y. (2023). Serum zinc and periodontitis in non-diabetic smoking and non-smoking adults: NHANES 2011–2014. Oral Dis..

[60] Pushparani D.S. (2016). Serum Zinc and β D Glucuronidase Enzyme Level in Type 2 Diabetes Mellitus with Periodontitis. Curr. Diabetes Rev..

[61] Sundaram G., Ramakrishnan T., Parthasarathy H., Moses J., Lalitha T. (2017). Evaluation of Micronutrient (Zinc, Magnesium, and Copper) Levels in Serum and Glycemic Status after Nonsurgical Periodontal Therapy in Type 2 Diabetic Patients with Chronic Periodontitis. Contemp. Clin. Dent..

[62] Yamori M., Njelekela M., Mtabaji J., Yamori Y., Bessho K. (2011). Hypertension, periodontal disease, and potassium intake in nonsmoking, nondrinker african women on no medication. Int. J. Hypertens..

[63] Thomas B., Kumari S., Ramitha K., Ashwini Kumari M.B. (2010). Comparative evaluation of micronutrient status in the serum of diabetes mellitus patients and healthy individuals with periodontitis. J. Indian Soc. Periodontol..

[64] Thomas B., Ramesh A., Suresh S., Prasad B.R. (2013). A comparative evaluation of antioxidant enzymes and selenium in the serum of periodontitis patients with diabetes mellitus type 2. Contemp. Clin. Dent..

[65] Kim H.S., Park J.A., Na J.S., Lee K.H., Bae K.H. (2014). Association between plasma levels of manganese and periodontal status: A study based on the fourth Korean National Health and Nutrition Examination Survey. J Periodontol..

[66] Varela-López A., Giampieri F., Bullón P., Battino M., Quiles J.L. (2016). A Systematic Review on the Implication of Minerals in the Onset, Severity and Treatment of Periodontal Disease. Molecules.

[67] Huang Z., Rose A.H., Hoffmann P.R. (2012). The role of selenium in inflammation and immunity: From molecular mechanisms to therapeutic opportunities. Antioxid. Redox Signal..

[68] Wu F., Xu K., Liu L., Zhang K., Xia L., Zhang M., Teng C., Tong H., He Y., Xue Y. (2021). Corrigendum: Vitamin B12 Enhances Nerve Repair and Improves Functional Recovery After Traumatic Brain Injury by Inhibiting ER Stress-Induced Neuron Injury. Front. Pharmacol..

[69] Mustafa M., Zarrough A., Bolstad A.I., Lygre H., Mustafa K., Hasturk H., Serhan C., Kantarci A., Van Dyke T.E. (2013). Resolvin D1 protects periodontal ligament. Am. J. Physiol. Cell Physiol..

[70] Laky M., Bertl K., Haririan H., Andrukhov O., Seemann R., Volf I., Assinger A., Gruber R., Moritz A., Rausch-Fan X. (2017). Serum levels of 25-hydroxyvitamin D are associated with periodontal disease. Clin. Oral Investig..

[71] Hiremath V.P., Rao C.B., Naik V., Prasad K.V. (2013). Anti-inflammatory effect of vitamin D on gingivitis: A dose-response randomised control trial. Oral Health Prev. Dent..

[72] Pappe C.L., Lutzenberger S., Goebler K., Meier S., Jeitler M., Michalsen A., Dommisch H. (2024). Effect of a Whole-Food Plant-Based Diet on Periodontal Parameters in Patients With Cardiovascular Risk Factors: A Secondary Sub-Analysis of a Randomized Clinical Trial. J. Clin. Periodontol..

